# Supplementary material for: Regulation of p53 Activity by (+)-Epiloliolide Isolated from Ulva lactuca
Source: Mar Drugs. 2021 Aug 5;19(8):450. doi: 10.3390/md19080450 (PMC8399812; doi:10.3390/md19080450)
Supplement: Supplementary file 1 [file marinedrugs-19-00450-s001.zip › marinedrugs-1297990-supplementary.pdf]

marine drugs

Supporting Information

Regulation of p53 Activity by (+)-Epiloliolide Isolated from *Ulva lactuca*

Yuheon Chung [1], Seula Jeong [1], In-Kyoung Lee [2], Bong-Sik Yun [2], Jung Sup Lee [3], Seungil Ro [4] and Jong Kun Park [1,*]

[1] Division of Biological Science, Wonkwang University, Iksan 54538, Korea; yhchung@wku.ac.kr (Y.C.); sla0940@wku.ac.kr (S.J.)

[2] Division of Biotechnoloy and Advanced Institute of Environment and Bioscience, College of Environmental and Bioresource Sciences, Jeonbuk National University, Iksan 54596, Korea; iklee@jbnu.ac.kr (I.-K.L.); bsyun@jbnu.ac.kr (B.-S.Y.)

[3] Department of Life Science, Chosun University, Gwangju 61452, Korea; jsplee@chosun.ac.kr

[4] Department of Physiology and Cell Biology, University of Nevada School of Medicine, Reno, NV 89557, USA; sro@med.unr.edu(R.S.)

* Correspondence: jkpark@wku.ac.kr; Tel.: +82-10-2936-2781; Fax: +82-63-843-2781

Table of Contents

Figure S1. ^{1}H NMR spectrum of (+)-epiloliolide.
Figure S2. ^{13}C NMR spectrum of (+)-epiloliolide.
Figure S3. ^{1}H-^{1}H COSY spectrum of (+)-epiloliolide.
Figure S4. HMQC spectrum of (+)-epiloliolide.
Figure S5. HMBC spectrum of (+)-epiloliolide.
Figure S6. NOE difference spectrum of (+)-epiloliolide.
Figure S7. ^{1}H (red) and ^{13}C (blue) peaks assignments of (+)-epiloliolide.
Figure S8. Two-dimensional NMR correlations of (+)-epiloliolide.
Figure S9. Expression of p-p53 on full length gels.
Figure S10. Expression of p53 on full length gels.
Figure S11. Expression of p21 on full length gels.
Figure S12. Expression of Bim on full length gels.
Figure S13. Expression of Bax on full length gels.
Figure S14. Expression of Bcl-2 on full length gels.

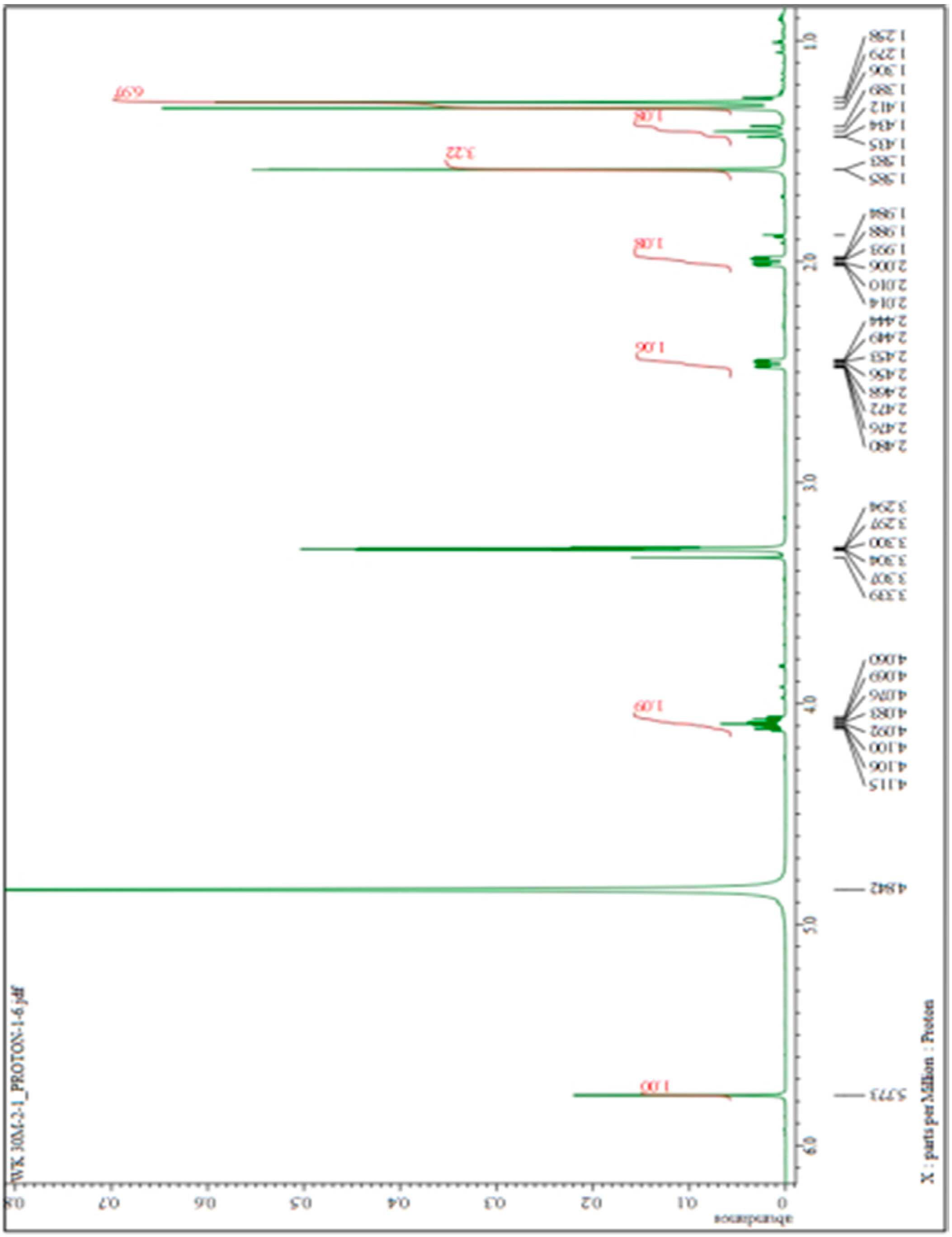

Figure S1. ^1H NMR spectrum of (+)-epiloliolide.

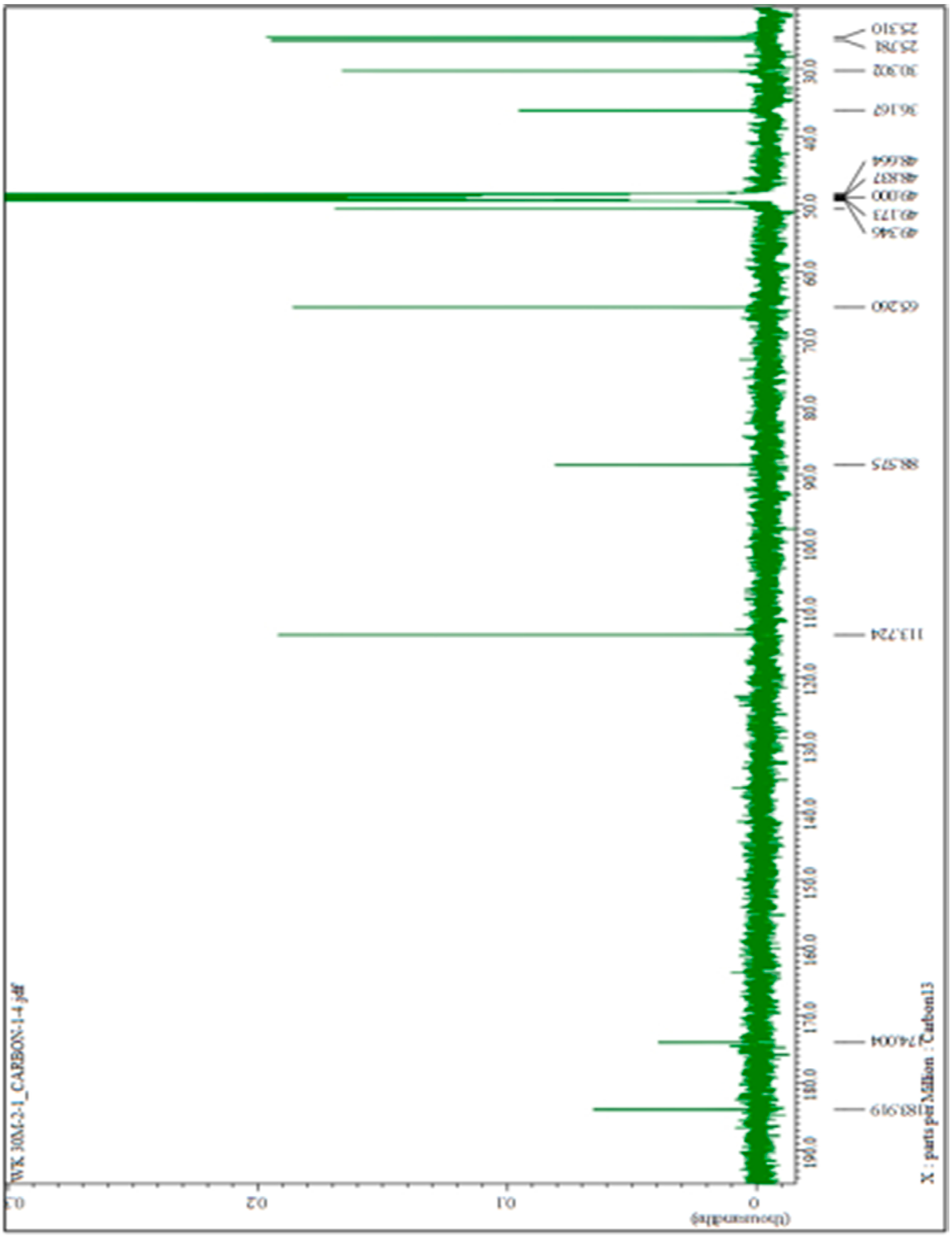

Figure S2. ^{13}C NMR spectrum of (+)-epiloliolide.

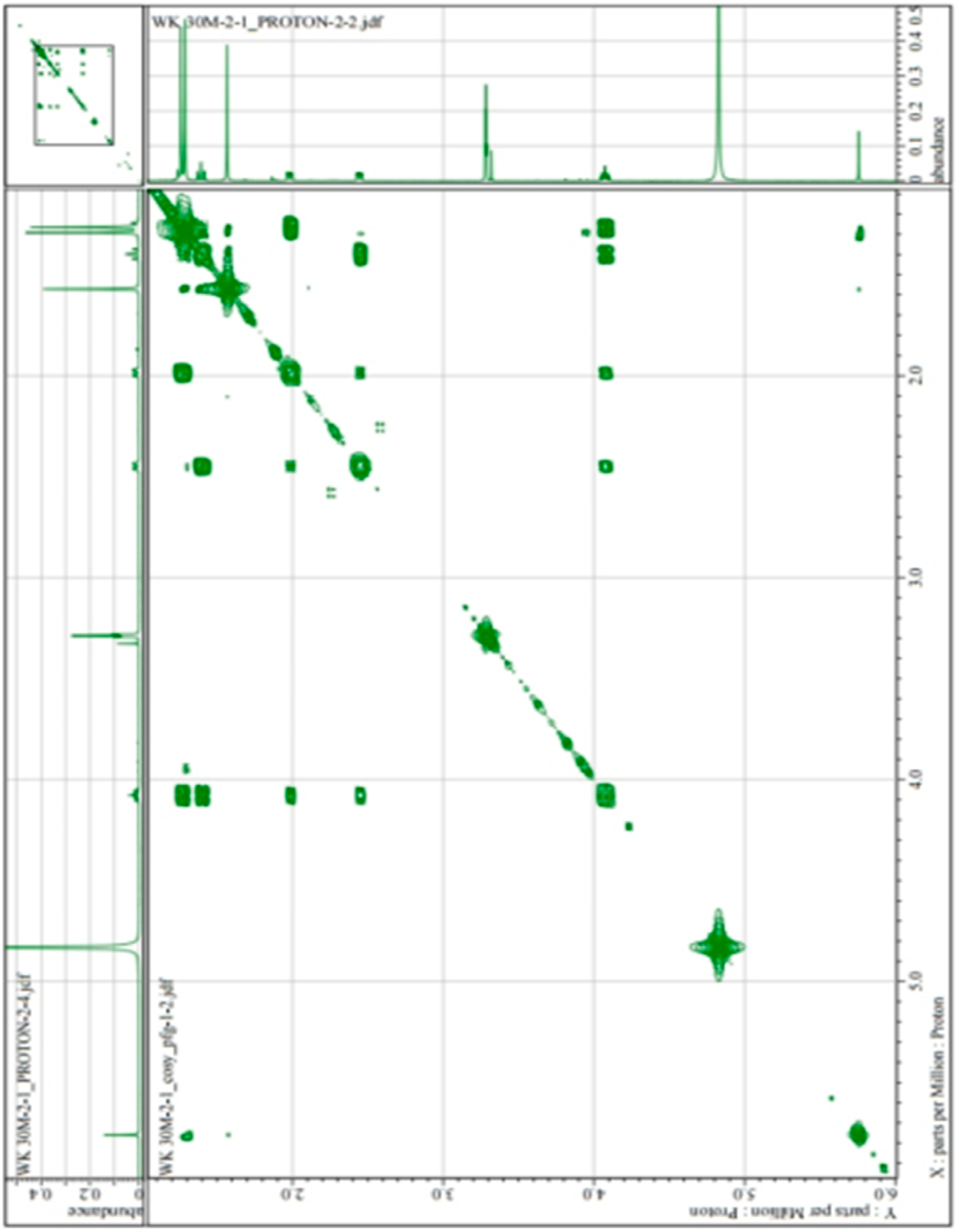

Figure S3. ^{1}H-^{1}H COSY spectrum of (+)-epiloliolide.

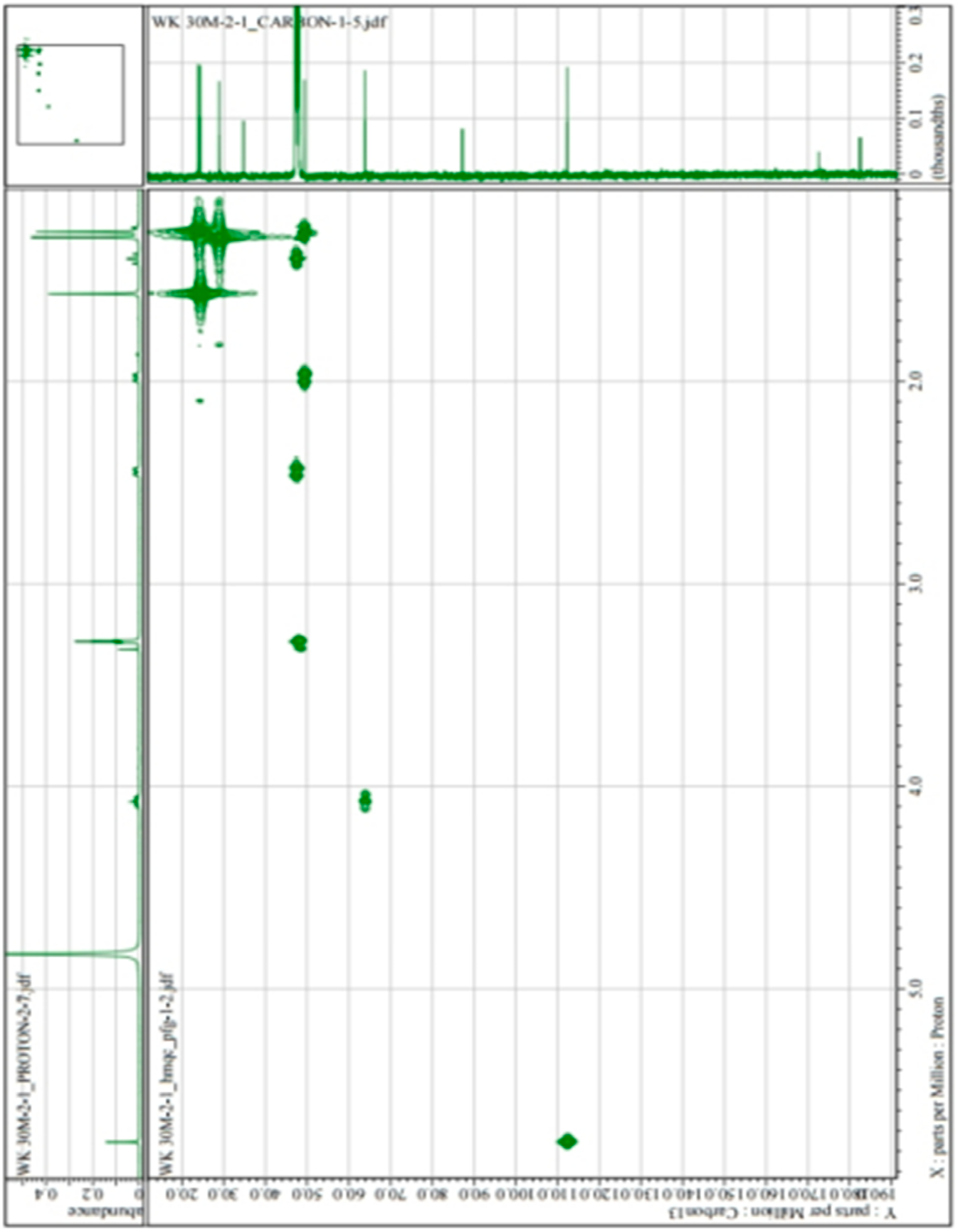

Figure S4. HMQC spectrum of (+)-epiloliolide.

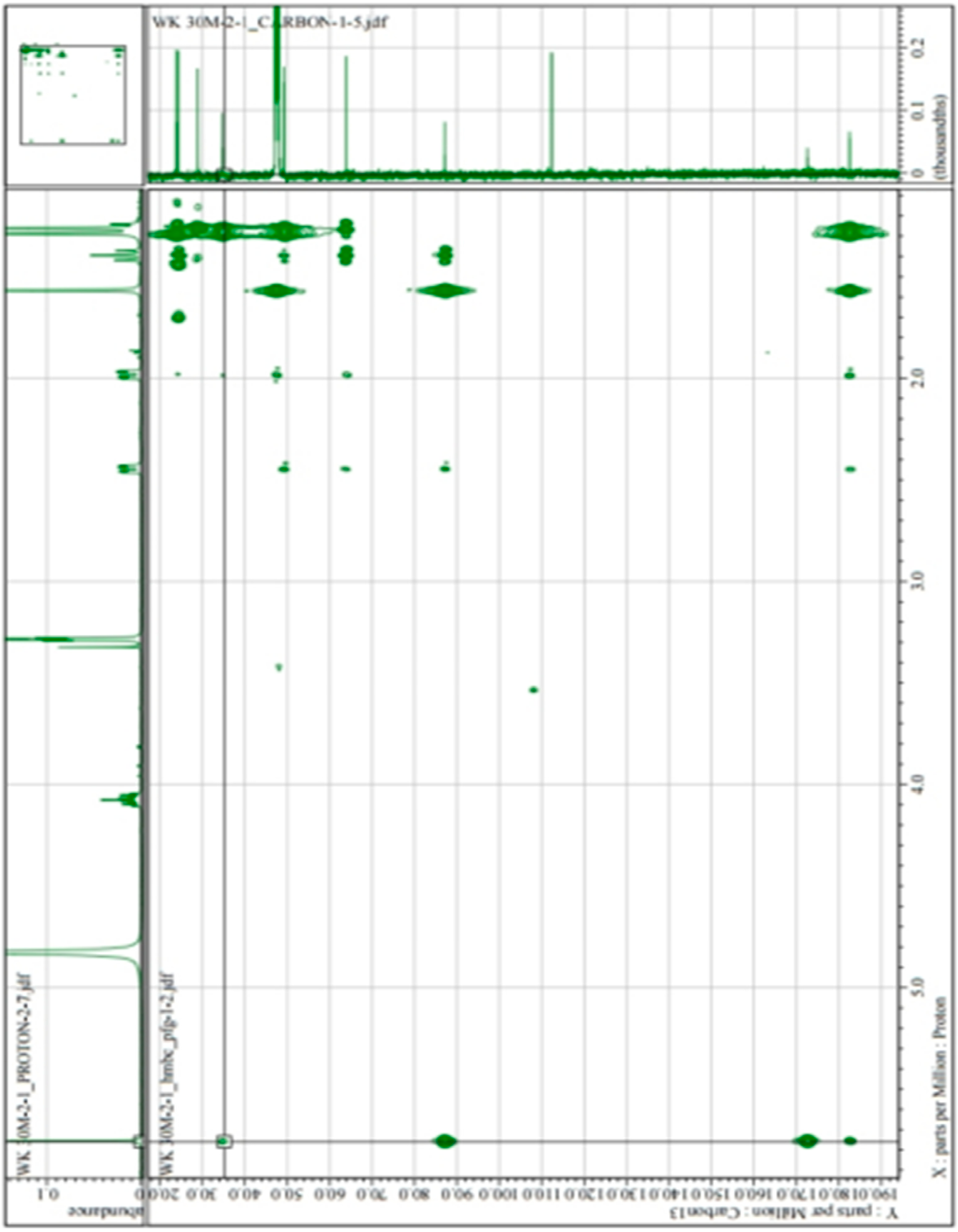

Figure S5. HMBC spectrum of (+)-epiloliolide.

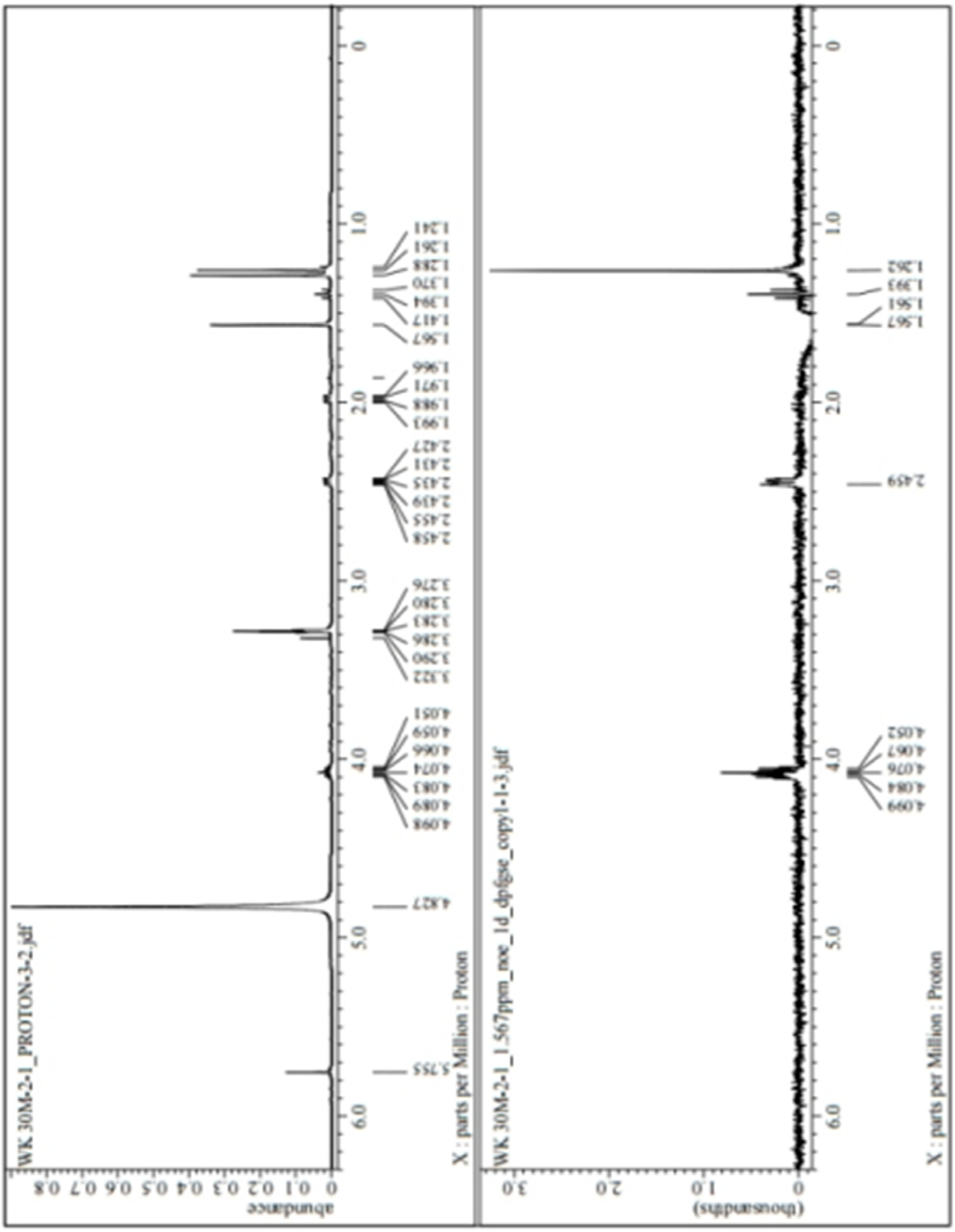

Figure S6. NOE difference spectrum of (+)-epiloliolide.

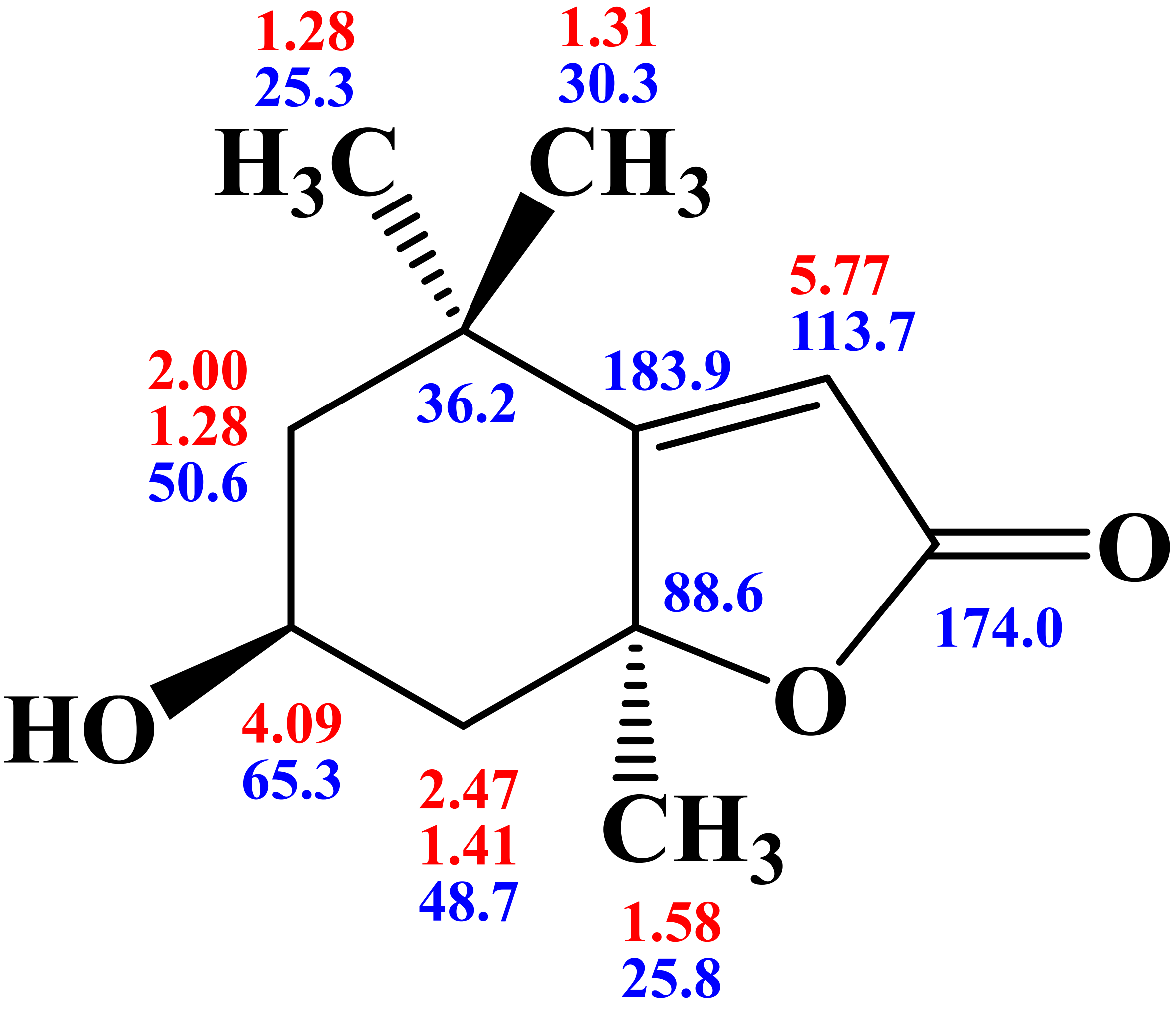

Figure S7. [1]H (red) and [13]C (blue) peaks assignments of (+)-epiloliolide.

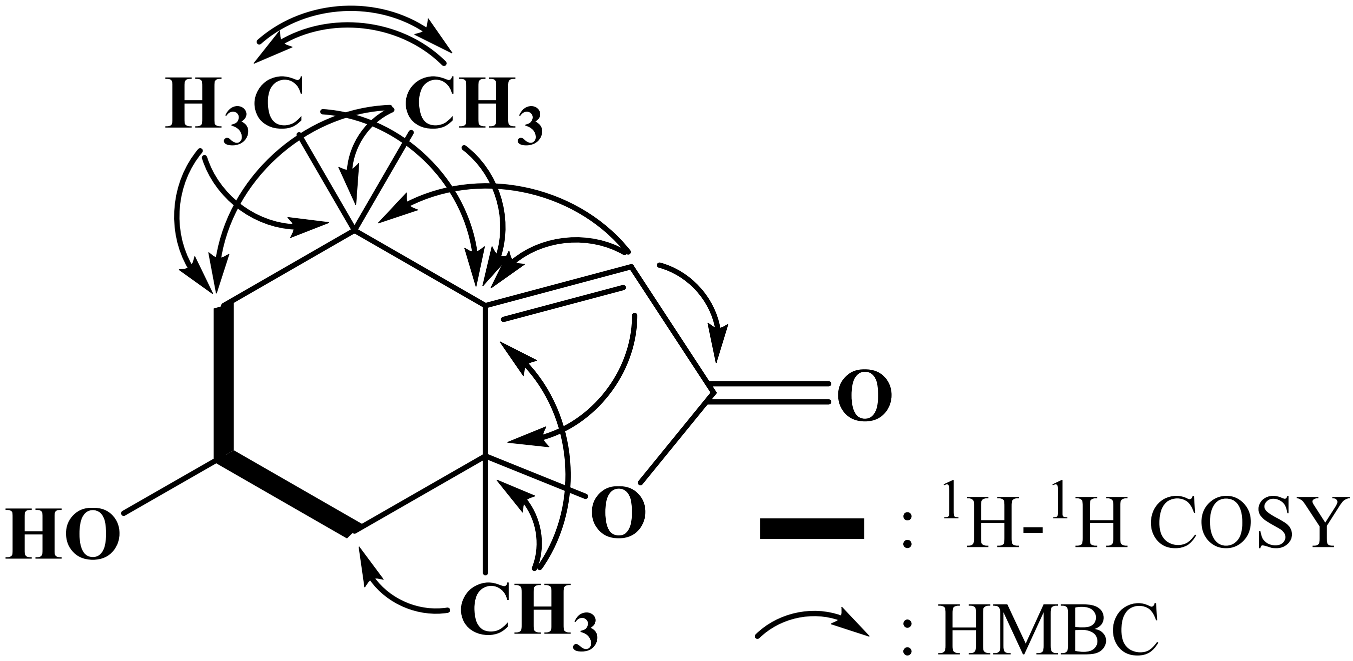

Figure S8. Two-dimensional NMR correlations of (+)-epiloliolide.

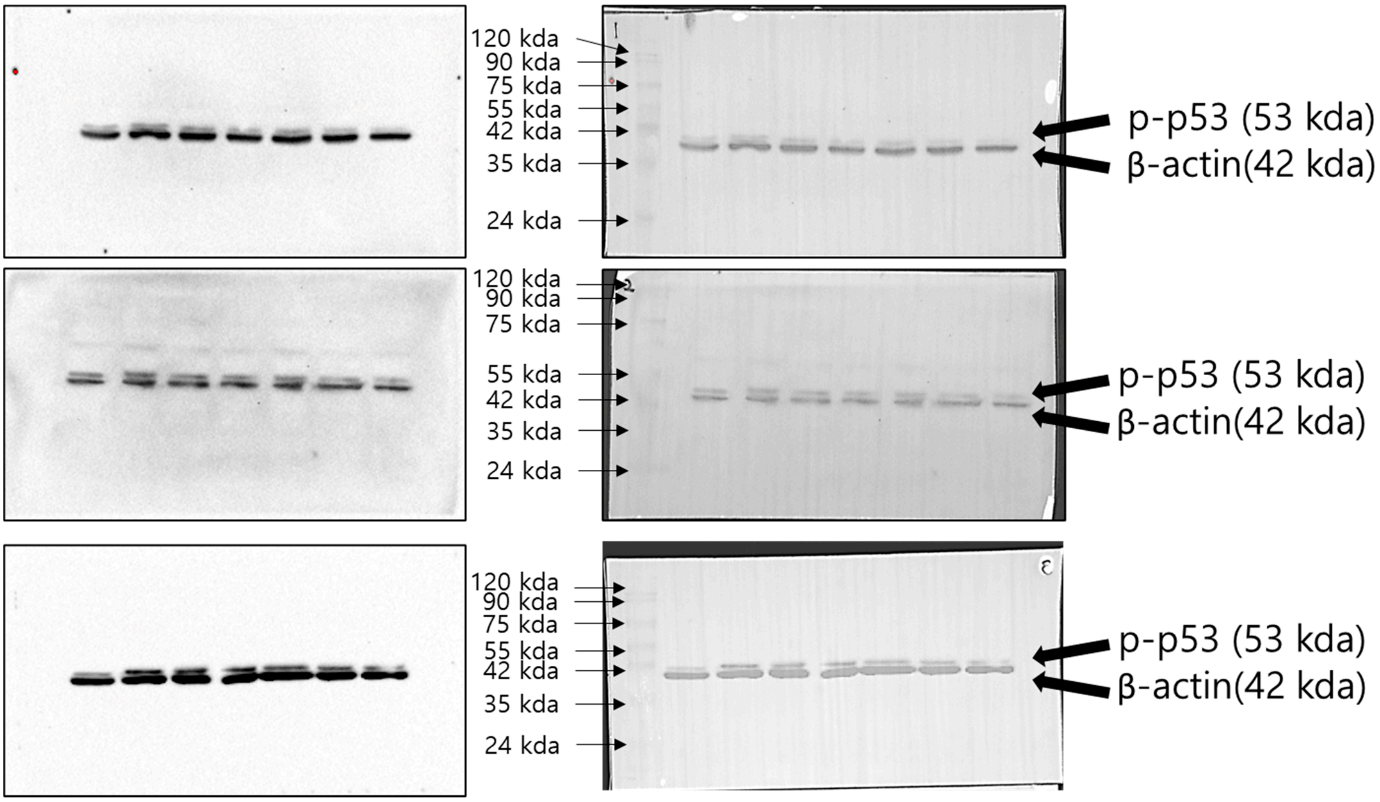

Figure S9. Expression of p-p53 on full length gels.

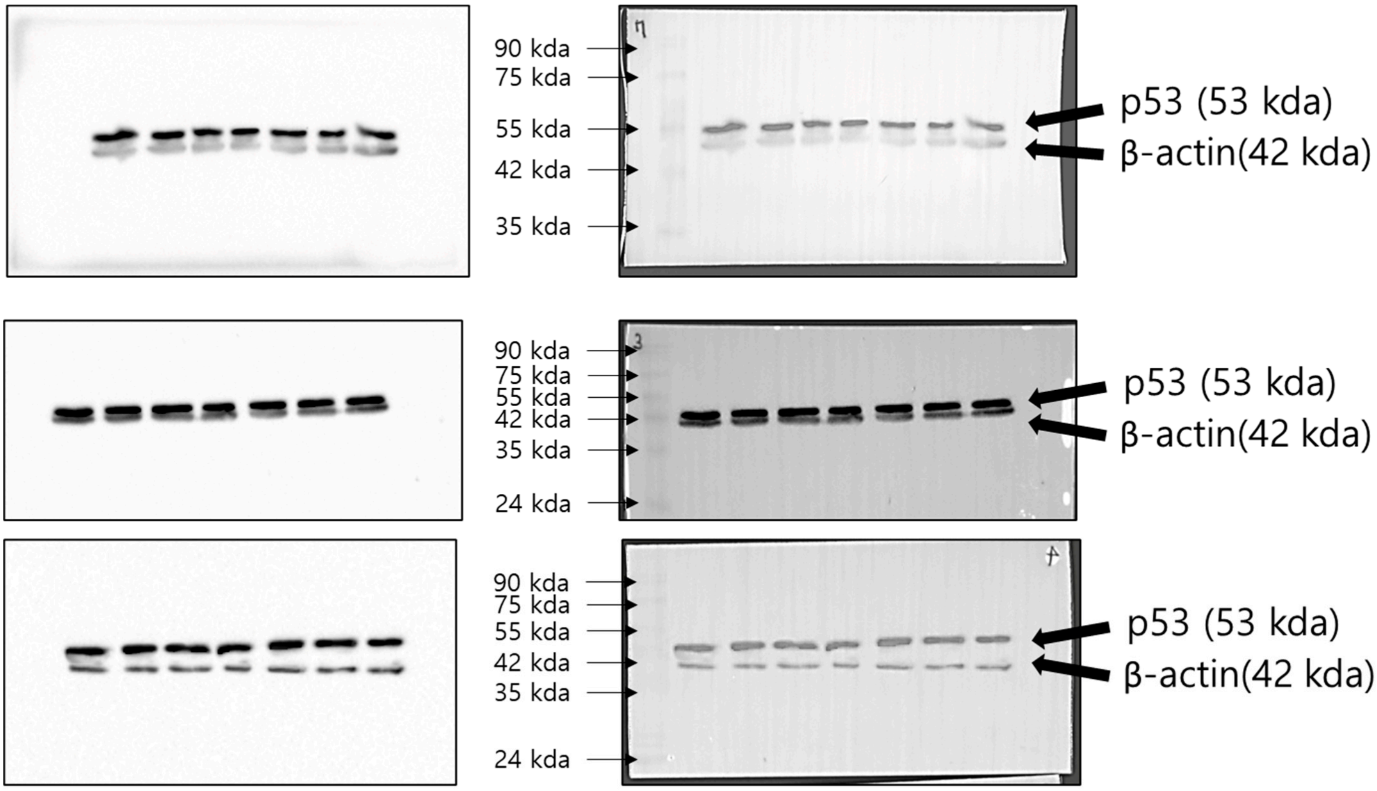

Figure S10. Expression of p53 on full length gels.

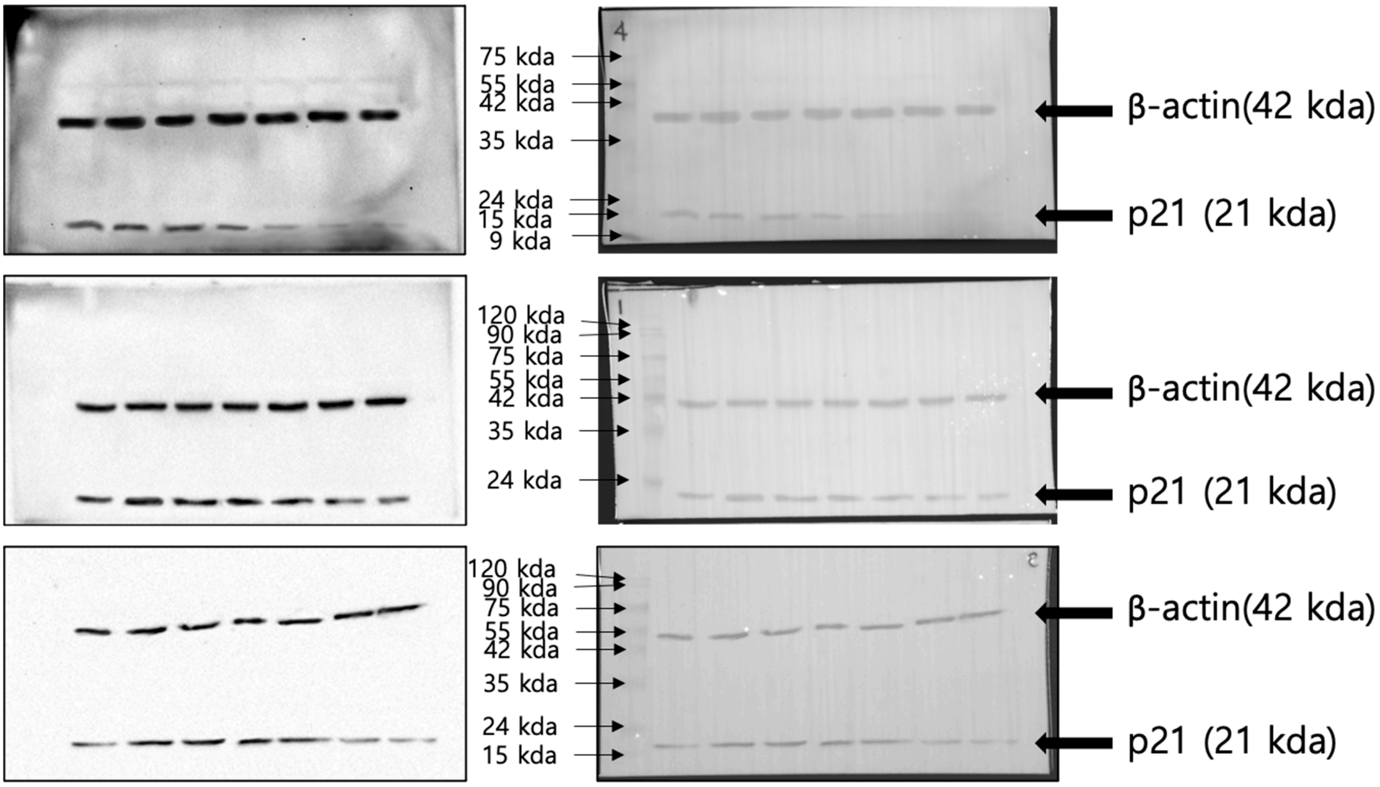

Figure S11. Expression of p21 on full length gels.

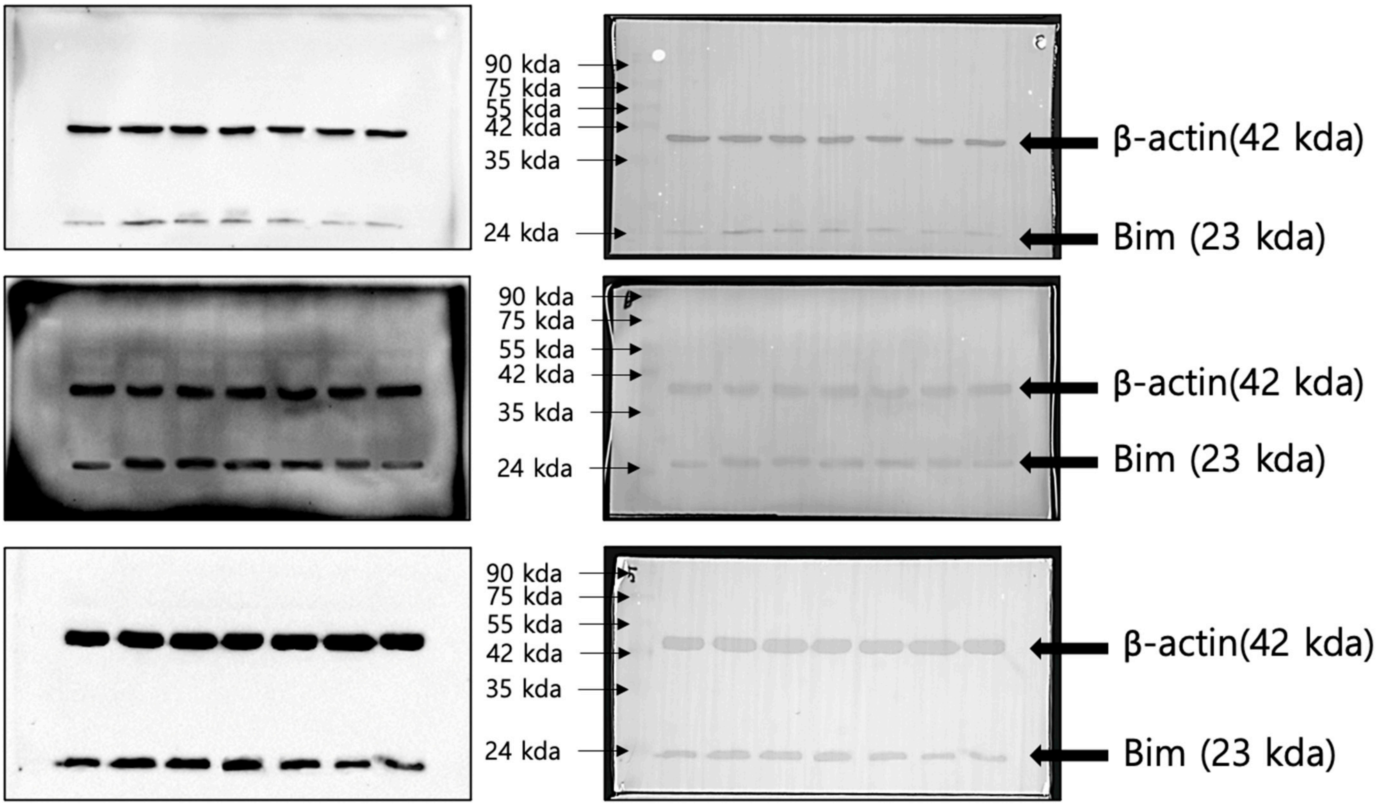

Figure S12. Expression of Bim on full length gels.

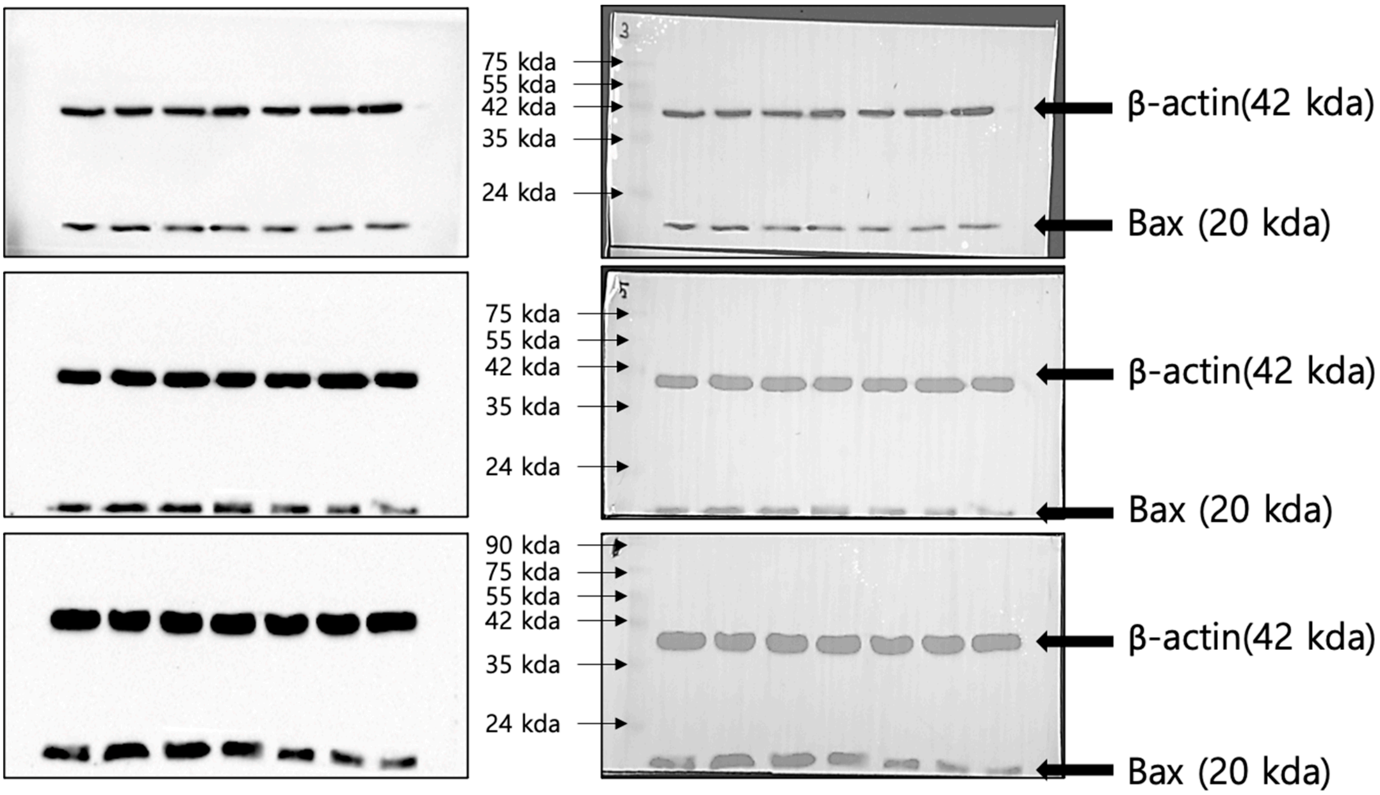

Figure S13. Expression of Bax on full length gels.

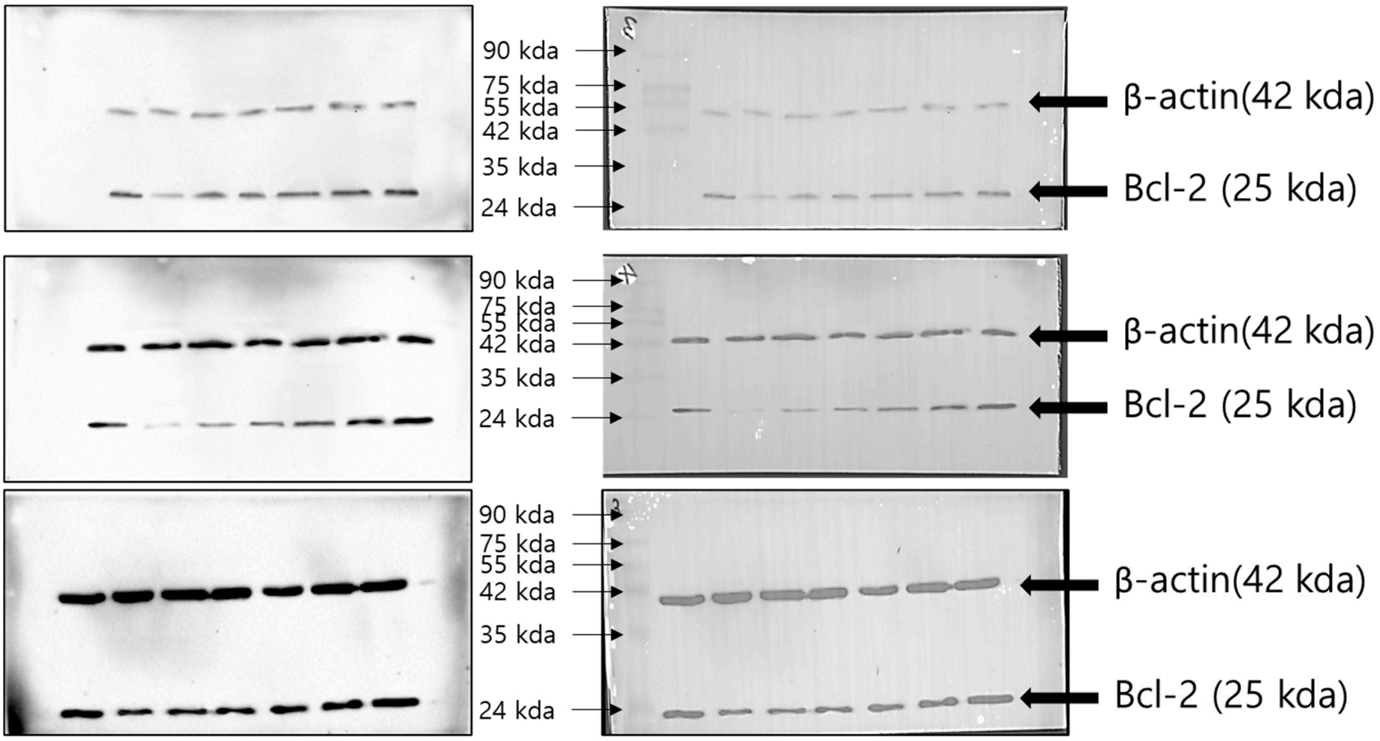

Figure S14. Expression of Bcl-2 on full length gels.
